# Supplementary figures and images for: Tonic ubiquitination of the central body weight regulator melanocortin receptor 4 (MC4R) promotes its constitutive exit from cilia
Source: PLoS Biol. 2025 Feb 3;23(2):e3003025. doi: 10.1371/journal.pbio.3003025 (PMC11825094; doi:10.1371/journal.pbio.3003025)

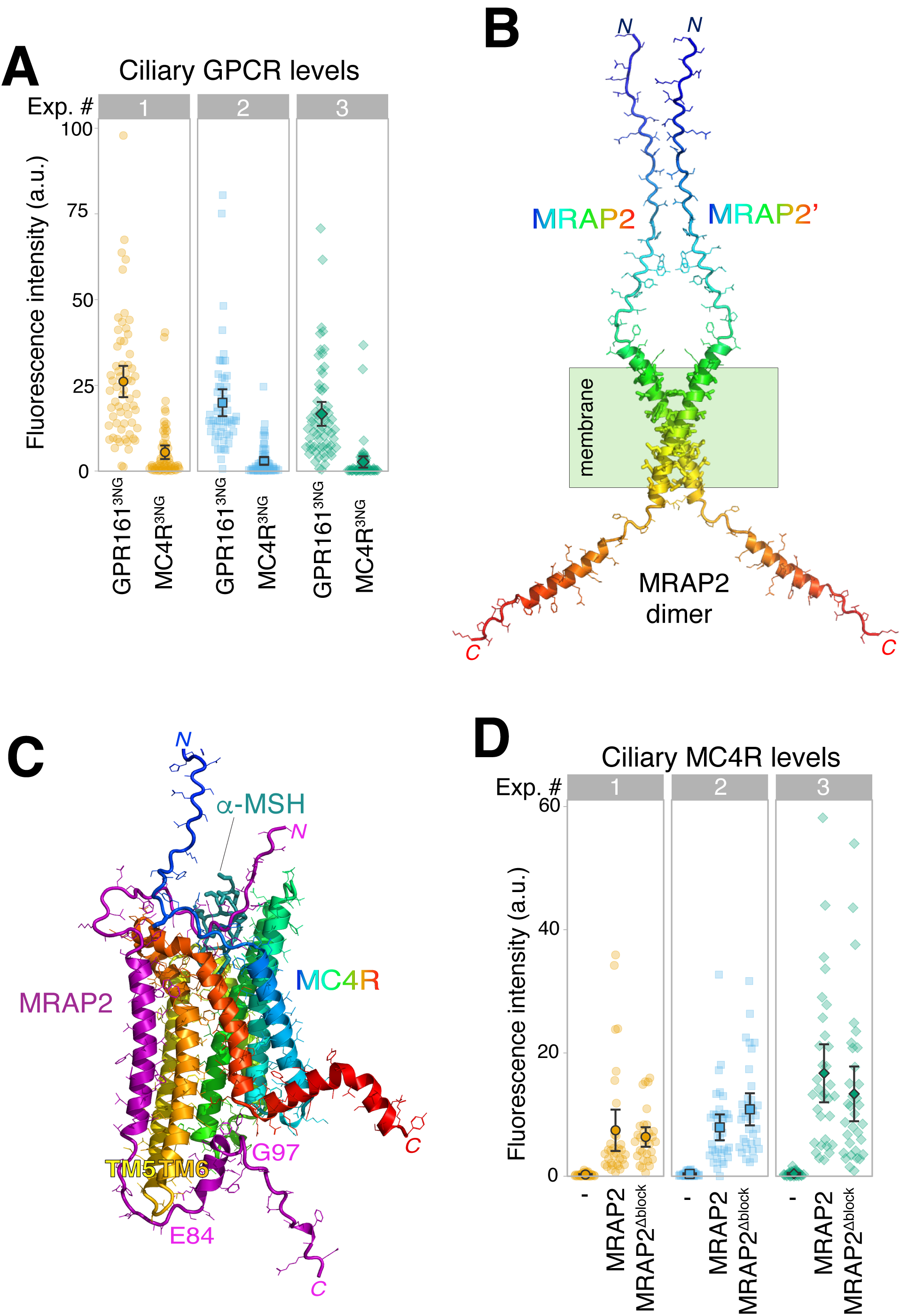

Supplement: S1 Fig — (A) Split values of the n = 3 independent experiments comparing the ciliary fluorescence intensity of GPR1613NG vs. MC4R3NG in Fig 1B. Data points belonging to each different experiment are encoded by translucent points of different color and shape. The average of each experiment is represented by solid points and the error bars represent 95% confidence interval. All underlying data are found S1 Data. (B) AlphaFold2.3 model of MRAP2 dimer. (C) AlphaFold2.3 model of human MC4R/MRAP2 in a 1:1:1 complex with human α-MSH. (D) Split values of the n = 3 independent experiments in Fig 4E comparing the MC4R3NG ciliary fluorescence intensity in IMCD3-[MC4R3NG] untransfected or transiently transfected cells with MRAP23FLAG or the MRAP2 version lacking the candidate MC4R inhibitory motif MRAP2Δblock-3FLAG. Data points belonging to each different experiment are encoded by translucent points of different color and shape. The average of each experiment is represented by solid points and the error bars represent 95% confidence interval. All underlying data are found S1 Data. (TIF) [file pbio.3003025.s001.tif]

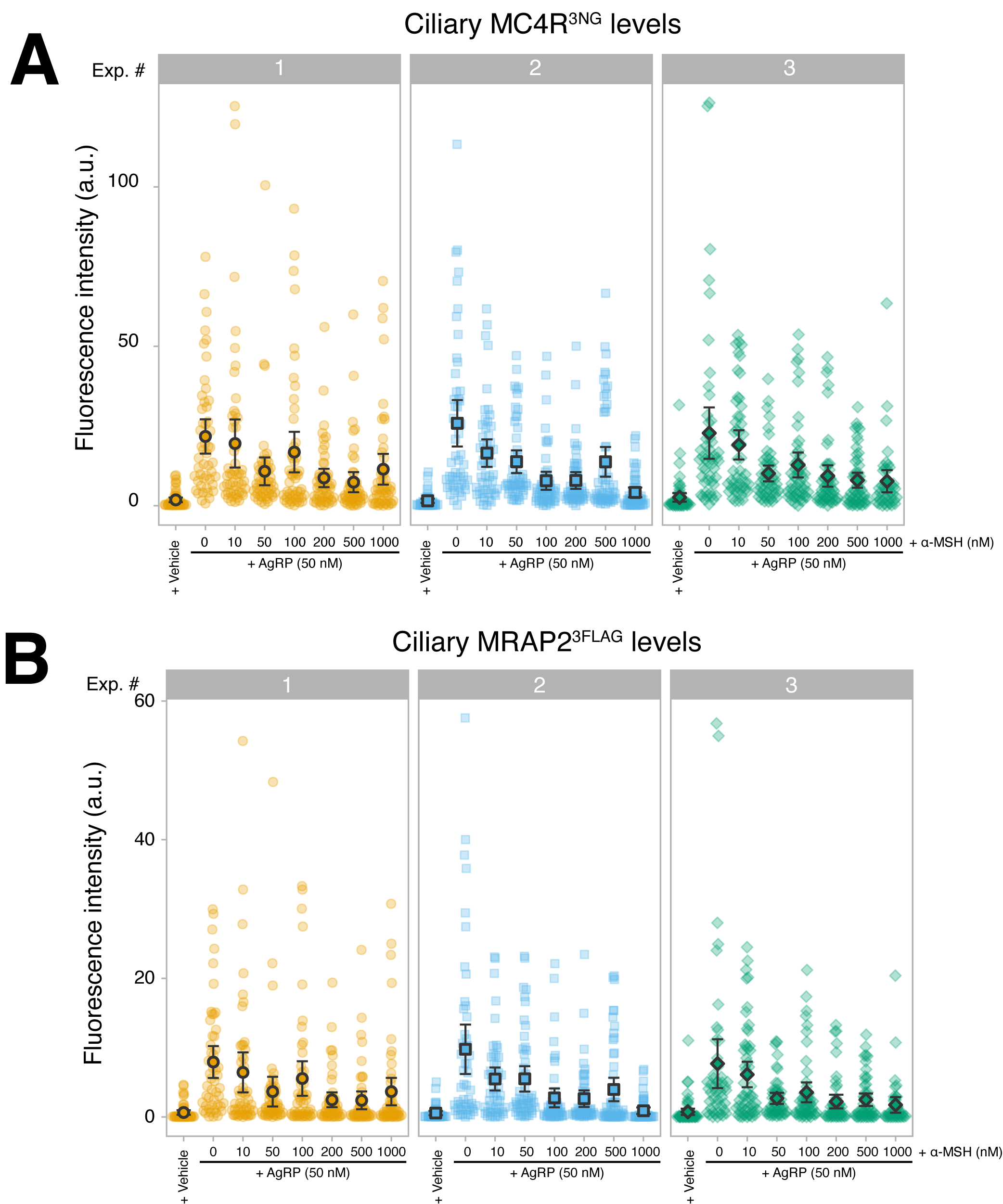

Supplement: S2 Fig — (A) Split values of the n = 3 independent experiments in Fig 3B comparing the MC4R3NG ciliary fluorescence intensity in IMCD3-[MRAP23FLAG;MC4R3NG] cells treated with either vehicle or 50 nM AgRP ± ⍺-MSH at different concentrations. Data points belonging to each different experiment are encoded by translucent points of different color and shape. The average of each experiment is represented by solid points and the error bars represent 95% confidence interval. All underlying data are found S1 Data. (B) Split values of the n = 3 independent experiments in Fig 3C comparing the MRAP23FLAG ciliary fluorescence intensity in IMCD3-[MRAP23FLAG;MC4R3NG] cells treated with either vehicle or 50 nM AgRP ± ⍺-MSH at different concentrations. Data points belonging to each different experiment are encoded by translucent points of different color and shape. The average of each experiment is represented by solid points and the error bars represent 95% confidence interval. All underlying data are found S1 Data. (TIF) [file pbio.3003025.s002.tif]

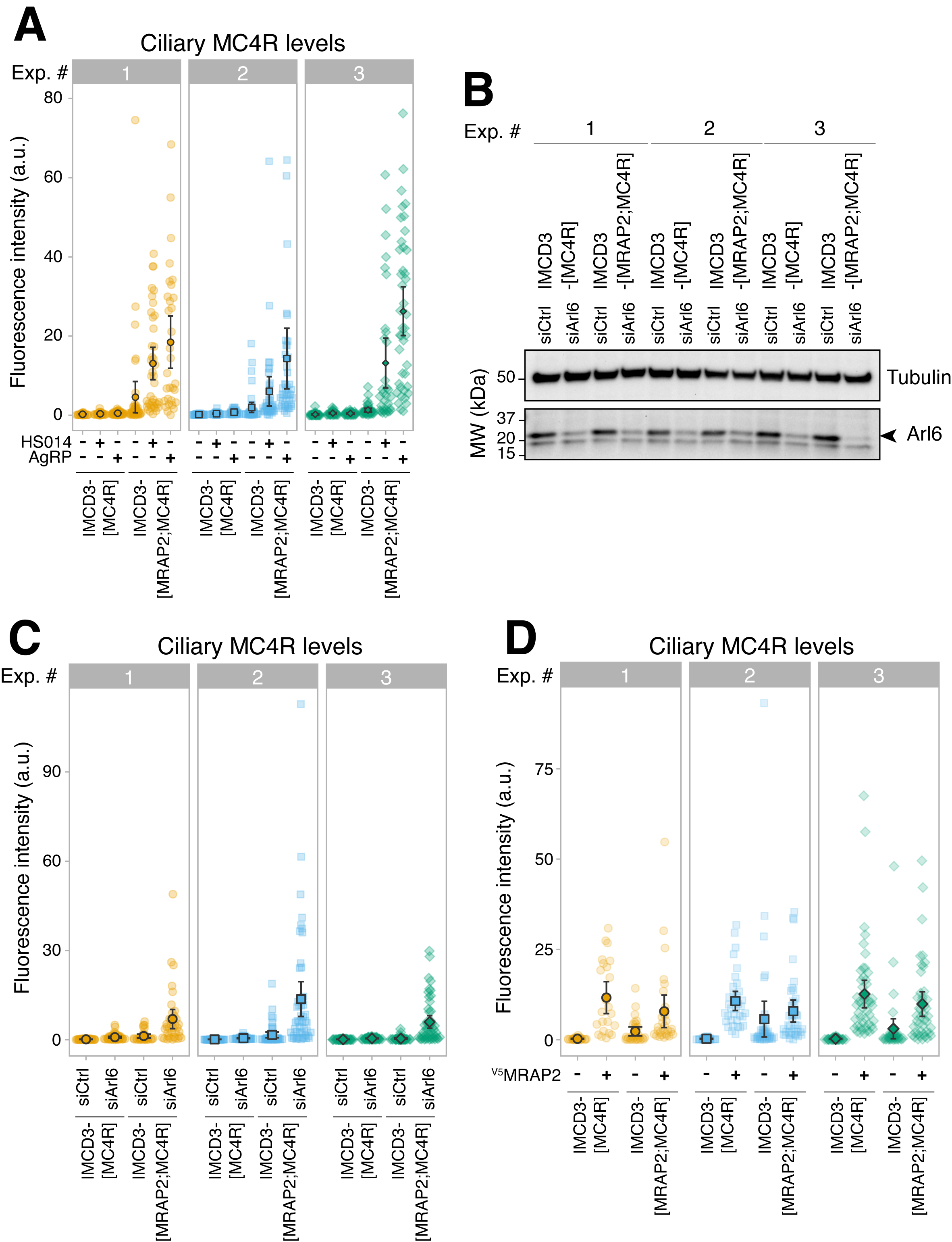

Supplement: S3 Fig — (A) Split values of the n = 3 independent experiments in Fig 5B comparing the MC4R3NG ciliary fluorescence intensity in cells expressing MC4R3NG alone or co-expressing MRAP23FLAG/MC4R3NG and treated with HS014, AgRP, or vehicle. Data points belonging to each different experiment are encoded by translucent points of different color and shape. The average of each experiment is represented by solid points and the error bars represent 95% confidence interval. All underlying data are found S1 Data. (B) Western blot of all n = 3 individual experiments showing proof of partial Arl6 knock-down after treatment with a siRNA targeted to Arl6 in both cells expressing MC4R3NG alone or co-expressing MRAP23FLAG/MC4R3NG. siRNA against luciferase (siLuc2) was used as a negative control. (C) Split values of the n = 3 independent experiments in Fig 5D comparing the MC4R3NG ciliary fluorescence intensity in cells knocked-down for Arl6. Data points belonging to each different experiment are encoded by translucent points of different color and shape. The average of each experiment is represented by solid points and the error bars represent 95% confidence interval. All underlying data are found S1 Data. (D) Split values of the n = 3 independent experiments in Fig 5F comparing the MC4R3NG ciliary fluorescence intensity in cells transiently transfected with V5MRAP2. Data points belonging to each different experiment are encoded by translucent points of different color and shape. The average of each experiment is represented by solid points and the error bars represent 95% confidence interval. All underlying data are found S1 Data. (TIF) [file pbio.3003025.s003.tif]

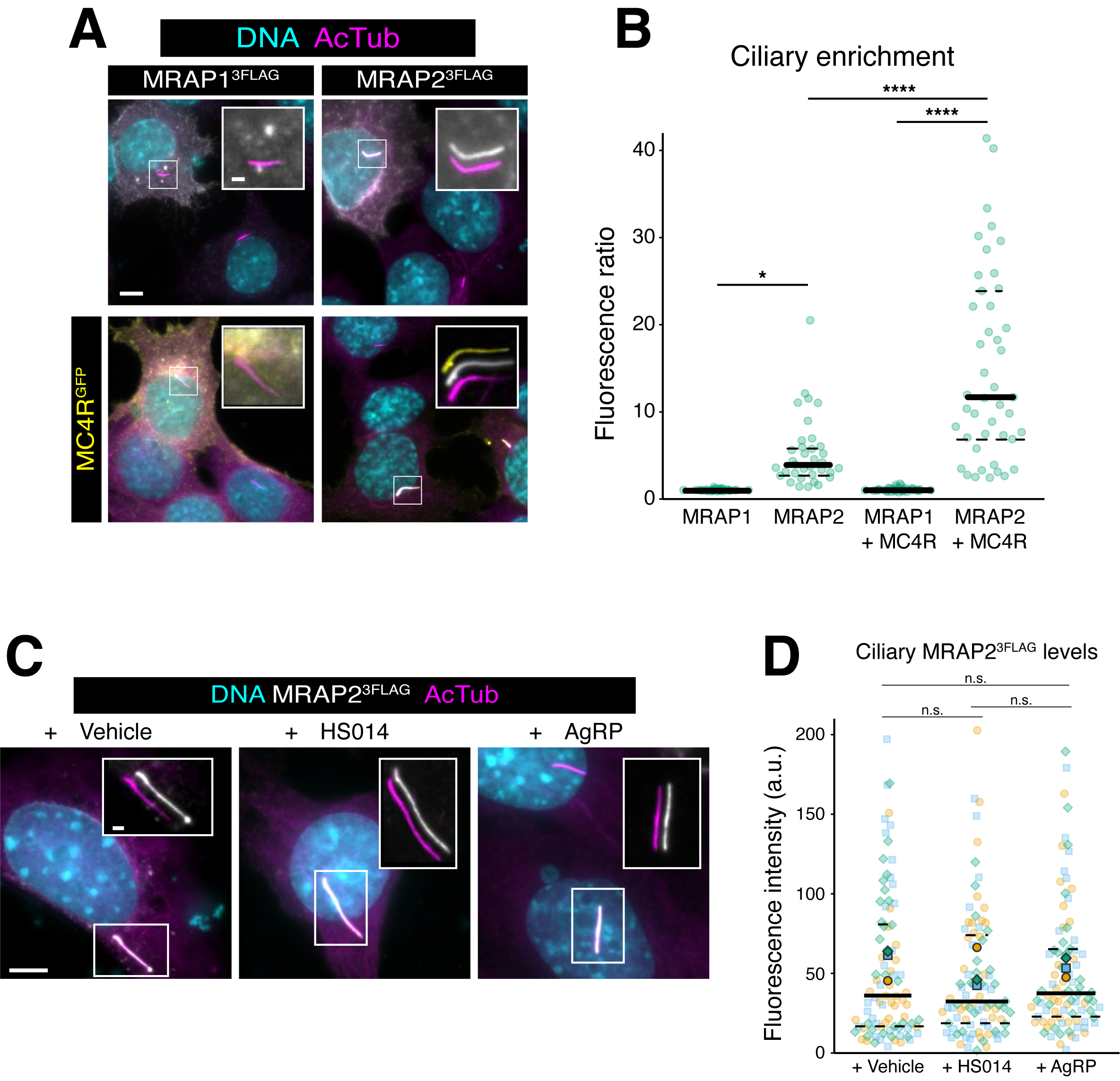

Supplement: S4 Fig — (A) Representative images of transiently transfected IMCD3 cells with either MRAP13FLAG or MRAP23FLAG and/or MC4RGFP. MC4RGFP was visualized through the intrinsic fluorescence of eGFP (yellow). Serum-starved cells were fixed and stained for acetylated tubulin (acTub, magenta), FLAG (MRAP1/23FLAG, white), and DNA (cyan). The white, yellow, and magenta channels are shifted to facilitate visualization of ciliary signals in the insets. Scale bars: 5 μm (main panel) and 1 μm (inset). (B) Plots of ciliary MRAP13FLAG or MRAP23FLAG transiently transfected alone or in combination with MC4RGFP in IMCD3 cells (n = 1). While MRAP1 does not localize to cilium, MRAP2 can localize to the primary cilium on its own. Co-expression of MC4R increases ciliary enrichment of MRAP2. The solid line represents the median; the dashed lines are used to represent the interquartile values. Asterisks indicate statistical significance values calculated by a one-way ANOVA on individual cilia followed by a Holm–Sidak post hoc test (* p < 0.05, **** p < 0.0001). All underlying data are found S1 Data. (C) Representative images of transiently transfected IMCD3 cells with MRAP23FLAG and subsequently treated with AgRP, HS014, or vehicle upon serum starvation for 24 h. Cells were fixed and stained for acetylated tubulin (acTub, magenta), FLAG (MRAP1/23FLAG, white), and DNA (cyan). The white and magenta channels are shifted to facilitate visualization of ciliary signals in the insets. Scale bars: 5 μm (main panel) and 1 μm (inset). (D) Superplot comparing the ciliary fluorescence intensity of MRAP23FLAG in transiently transfected IMCD3 cells and treated with AgRP, HS014, or vehicle. n = 3 independent experiments. Data points belonging to each different experiment are encoded by translucent points of different color and shape. The average of each experiment is represented by solid points. A solid line has been used to represent the median of global data and dashed lines to represent the interquartile values [file pbio.3003025.s004.tif]

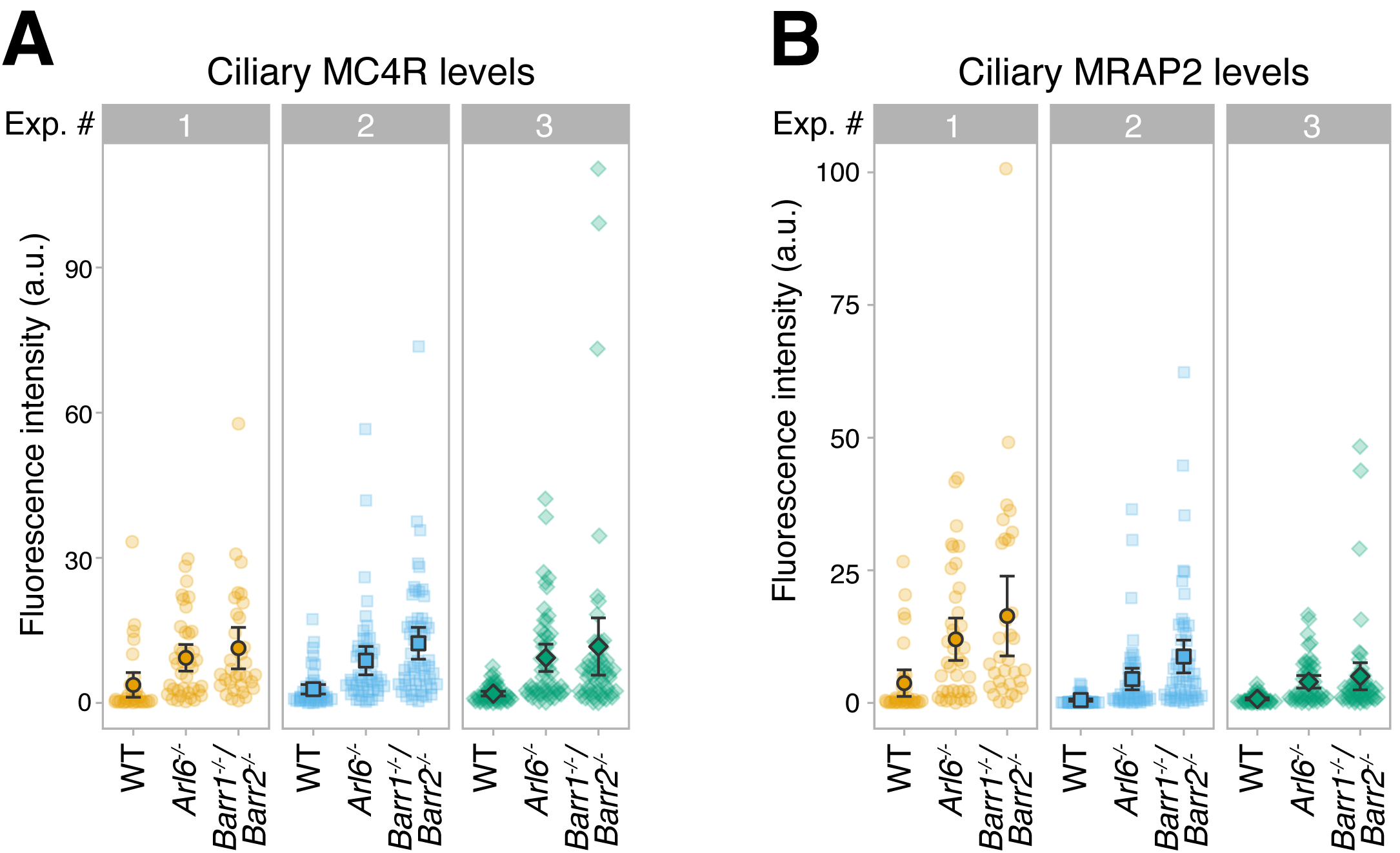

Supplement: S5 Fig — (A) Split values of the n = 3 independent experiments in Fig 6B comparing the MC4R3NG ciliary fluorescence intensity in cells co-expressing MRAP23FLAG/MC4R3NG in wild-type, Arl6-/- and Barr1-/-/Barr2-/- genetic backgrounds. Data points belonging to each different experiment are encoded by translucent points of different color and shape. The average of each experiment is represented by solid points and the error bars represent 95% confidence interval. All underlying data are found S1 Data. (B) Split values of the n = 3 independent experiments in Fig 6C comparing the MRAP23FLAG ciliary fluorescence intensity in cells co-expressing MRAP23FLAG/MC4R3NG in wild-type, Arl6-/- and Barr1-/-/Barr2-/- backgrounds. Data points belonging to each different experiment are encoded by translucent points of different color and shape. The average of each experiment is represented by solid points and the error bars represent 95% confidence interval. All underlying data are found S1 Data. (TIF) [file pbio.3003025.s005.tif]

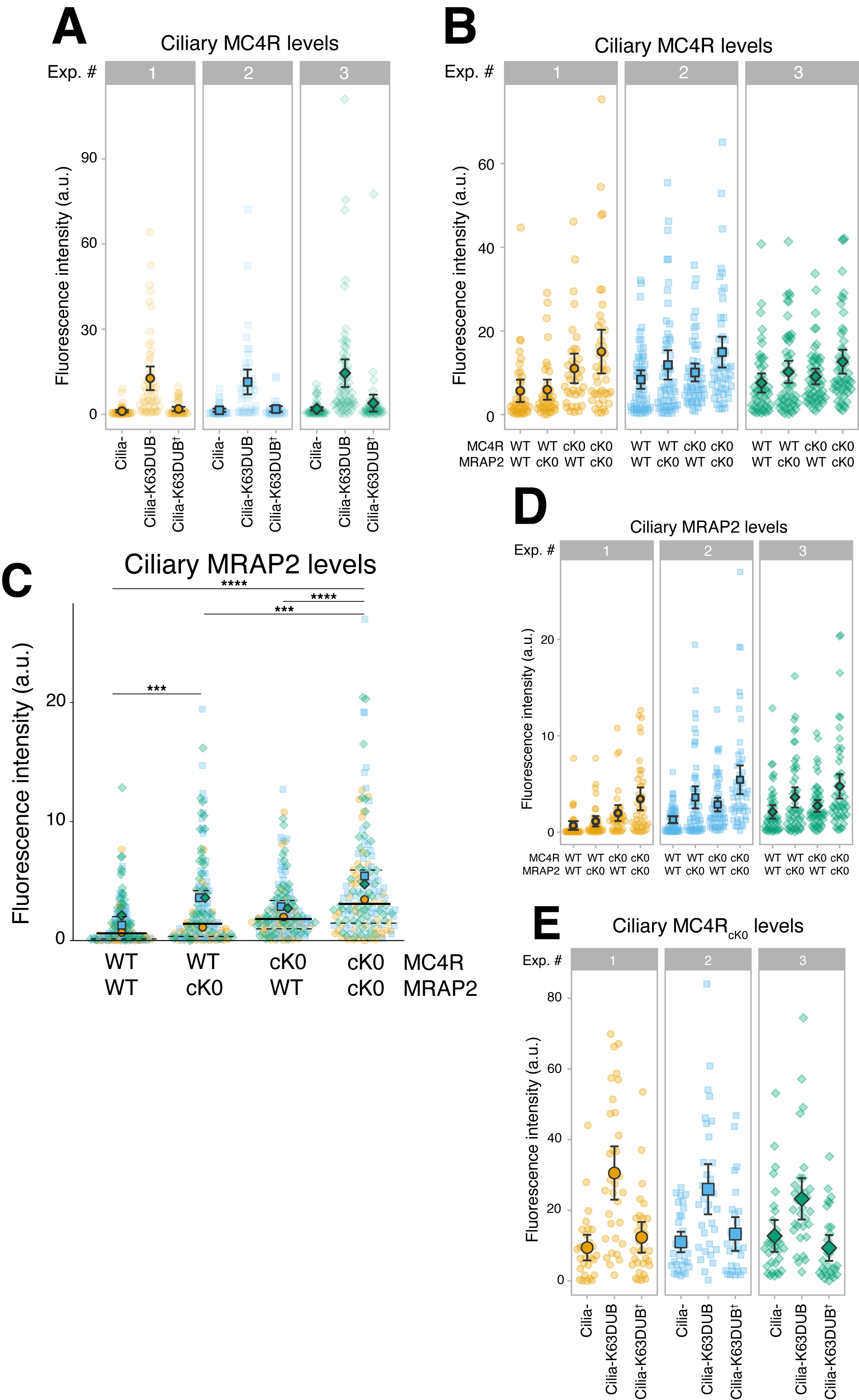

Supplement: S6 Fig — (A) Split values of the n = 3 independent experiments in Fig 8B comparing the ciliary fluorescence intensity of MC4R3NG in cells co-expressing MRAP23FLAG/MC4R3NG, transiently transfected with cilia-targeted K63-specific deubiquitinase catalytic domain of AMSH (cilia-K63DUB), the catalytically inactive counterpart (cilia-K63DUB†), or the cilia targeting sequence alone fused to the mScarlet reporter (cilia-reporter, CTS). Data points belonging to each different experiment are encoded by translucent points of different color and shape. The average of each experiment is represented by solid points and the error bars represent 95% confidence interval. All underlying data are found S1 Data. (B) Split values of the n = 3 independent experiments in Fig 8E comparing the ciliary fluorescence intensity of MC4R3NG in non-clonal IMCD3 lines stably co-expressing wild-type MRAP23FLAG/MC4R3NG (wt superscript) or the ubiquitination-refractory variants where the intracellular lysines have been substituted by arginine residues (K0 superscript). Data points belonging to each different experiment are encoded by translucent points of different color and shape. The average of each experiment is represented by solid points and the error bars represent 95% confidence interval. All underlying data are found S1 Data. (C) Superplot comparing the ciliary fluorescence intensity of MRAP23FLAG in non-clonal IMCD3 lines stably co-expressing wild-type MRAP23FLAG/MC4R3NG (wt superscript, serpentine schematic without empty circles), or the ubiquitination-refractory variants where the intracellular lysines have been substituted by arginine residues (K0 superscript, serpentine schematic with empty circles indicating the location of the lysine-to-arginine substitution). n = 3 independent experiments. Data points belonging to each different experiment are encoded by translucent points of different color and shape. The average of each experiment is represented by solid points. A solid line has been used to [file pbio.3003025.s006.tif]
