# Supplementary material for: Tonic ubiquitination of the central body weight regulator melanocortin receptor 4 (MC4R) promotes its constitutive exit from cilia
Source: PLoS Biol. 2025 Feb 3;23(2):e3003025. doi: 10.1371/journal.pbio.3003025 (PMC11825094; doi:10.1371/journal.pbio.3003025)
Supplement: S1 Raw Images — The uncropped immunoblots for Figs 2B, 2C, 4F, 6D, 7A, 7B, and S3B are shown. (PDF) [file pbio.3003025.s009.pdf]

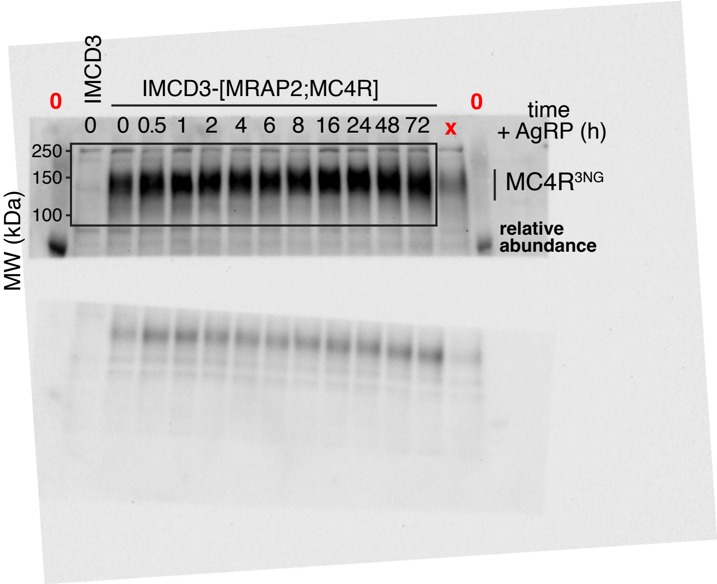

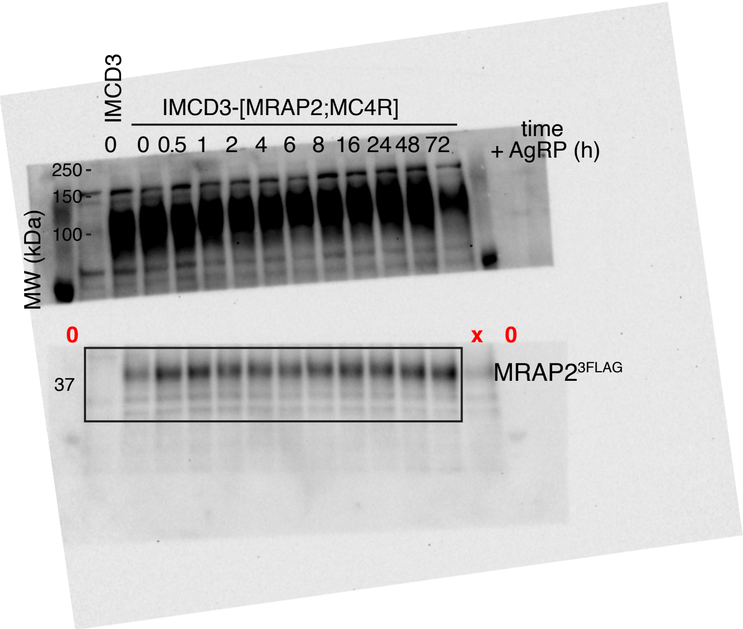

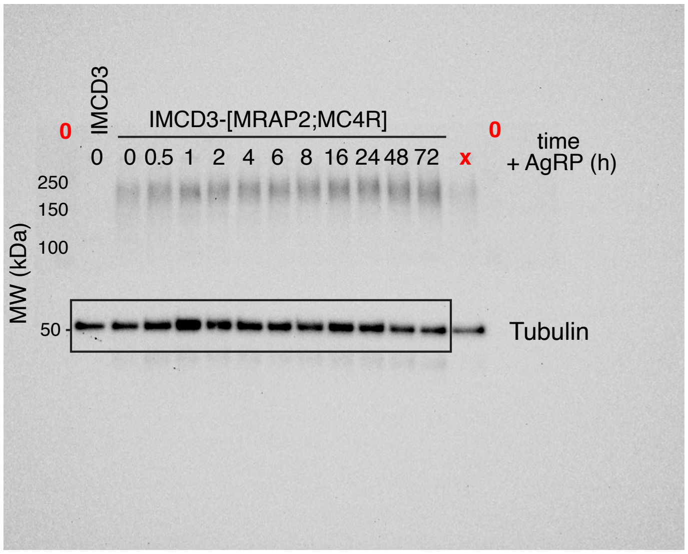

Three different exposures of the blot shown in **Fig. 2B**. The squared parts of the blot were used in the main figure.

The lane marked with an **x** was not shown in the main figure, and it corresponds to half the concentration of the sample *"IMCD3-[MRAP2;MC4R] at time 0"* .

The lanes marked with **0** contain molecular weight markers (Precision Plus Protein Standards Dual Color; Bio-Rad)

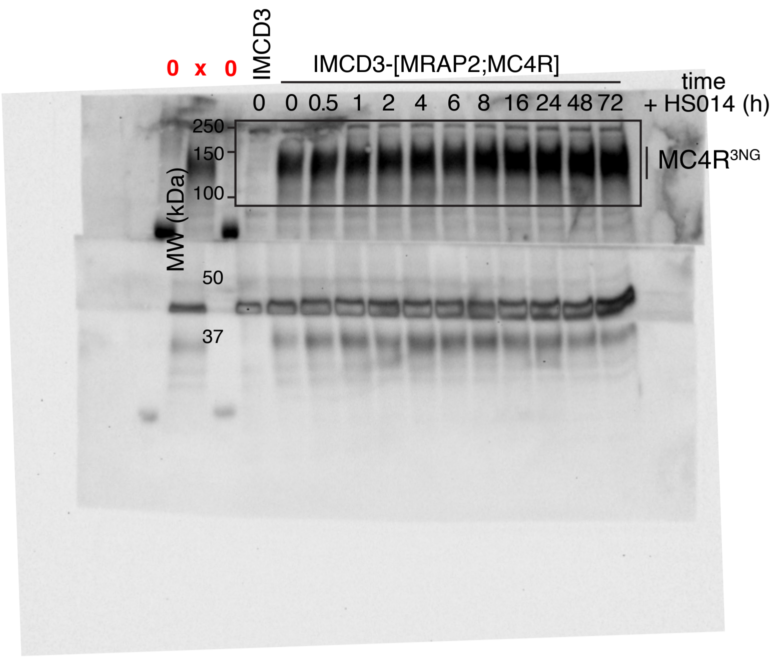

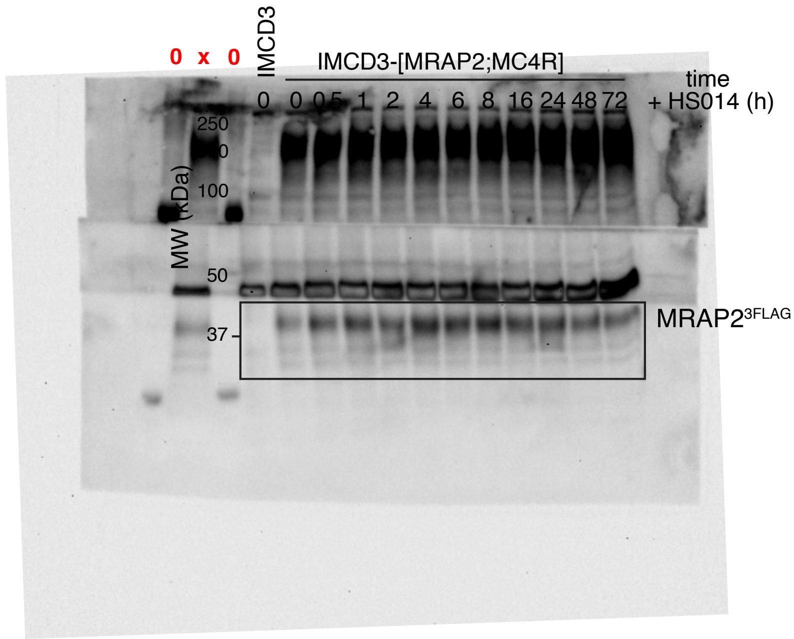

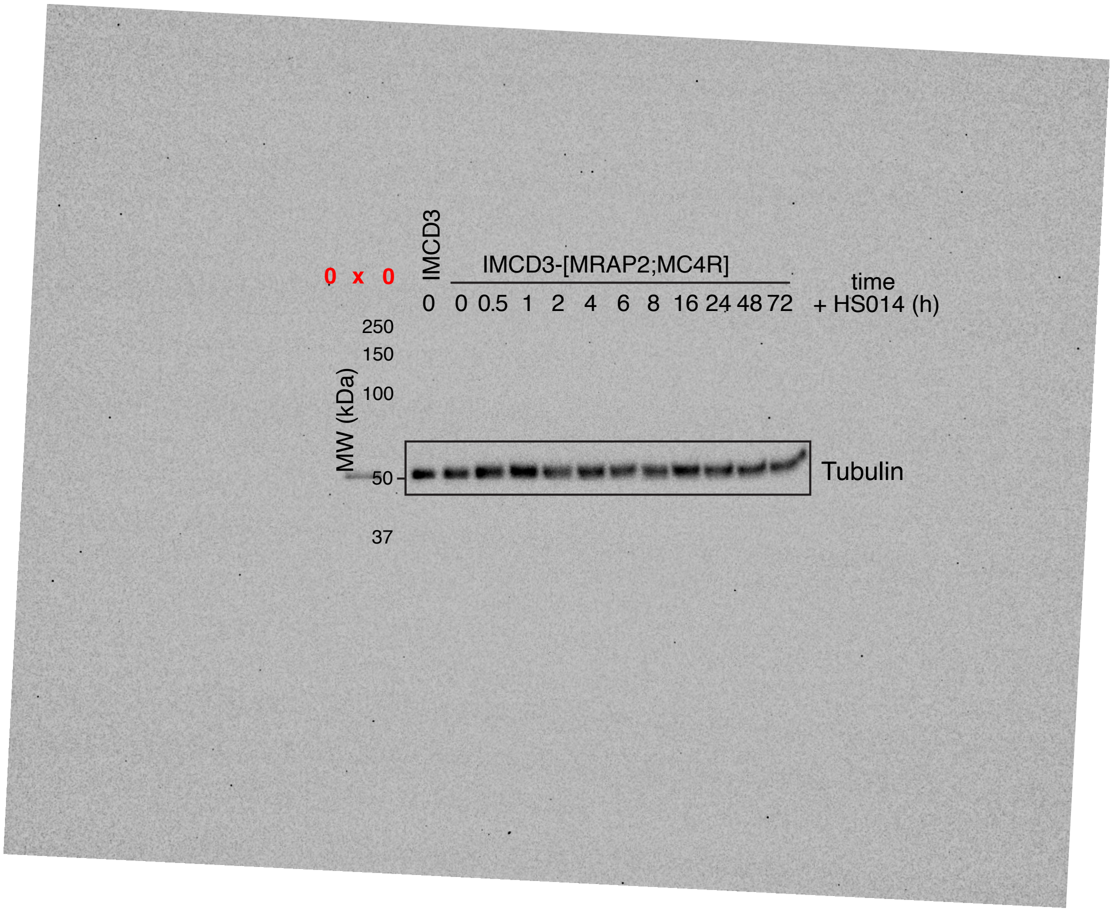

Three different exposures of the blot shown in the **Fig. 2C**. The squared parts of the blot were used in the main figure.

The lane marked with an **x** was not shown in the main figure, and it corresponds to half the concentration of the sample *“IMCD3-[MRAP2;MC4R] at time 0”*.

The lanes marked with **0** contain molecular weight markers (Precision Plus Protein Standards Dual Color; Bio-Rad)

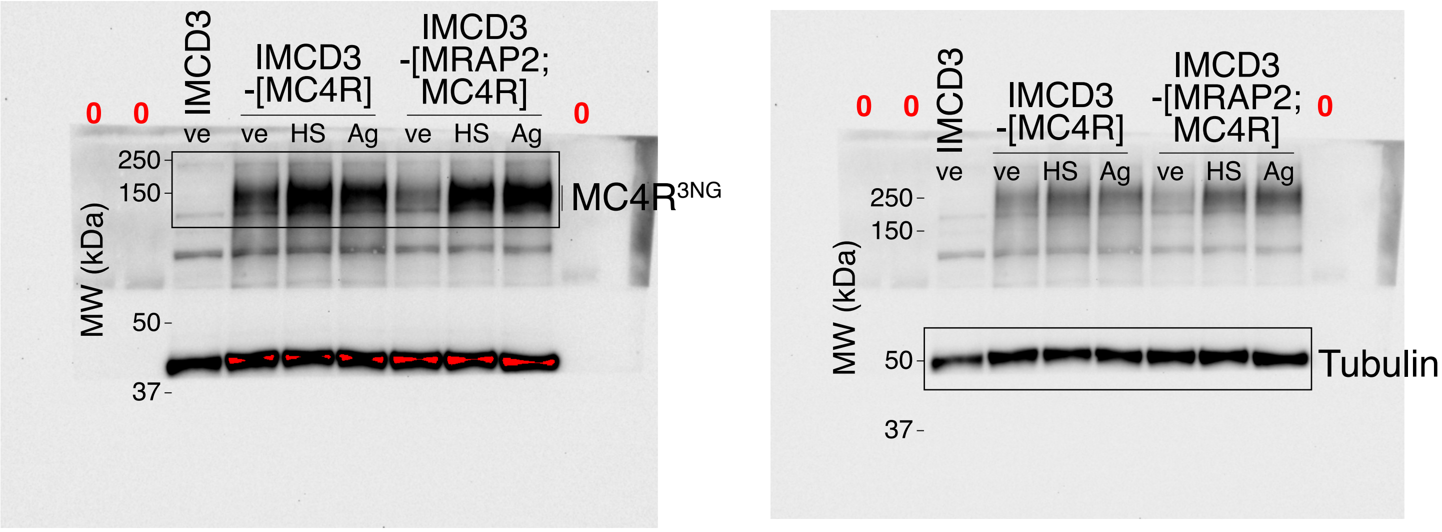

Two different exposures of the higher molecular weight portion of the blot shown in **Fig. 4F**. The squared parts of the blot were used in the main figure.

Bands appear red when the signal is saturated.

The lanes marked with **0** contain molecular weight markers (Precision Plus Protein Standards Dual Color; Bio-Rad)

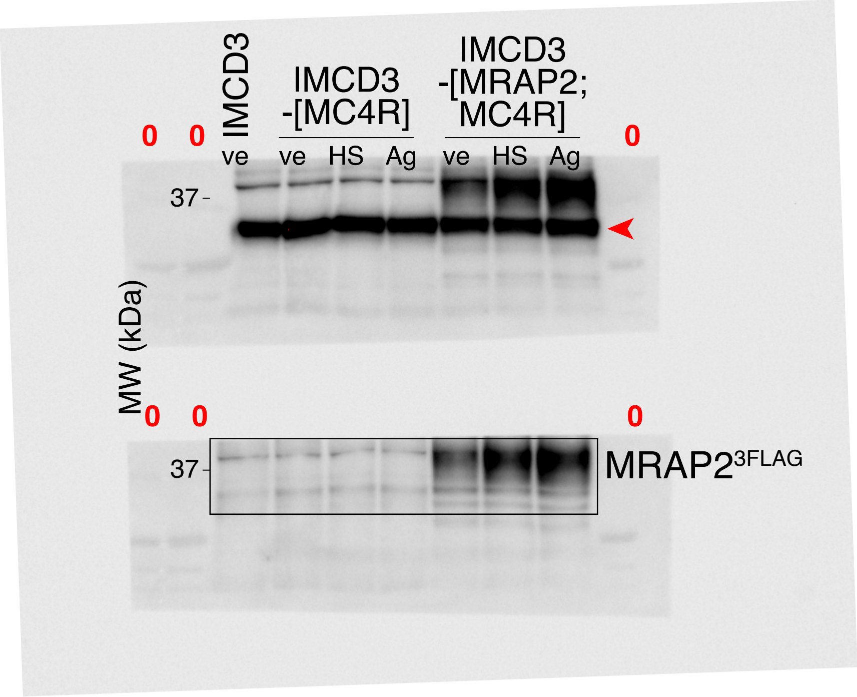

Two different blots containing the lower molecular weight portion of the blot shown in **Fig. 4F**. The squared part of the blot was used in the main figure.

The upper blot contains samples loaded in the absence of DTT. Note a strong unspecific band (red arrowhead).
The lower blot contains the same samples loaded in the presence of 5 mM DTT.

The lanes marked with **0** contain molecular weight markers (Precision Plus Protein Standards Dual Color; Bio-Rad)

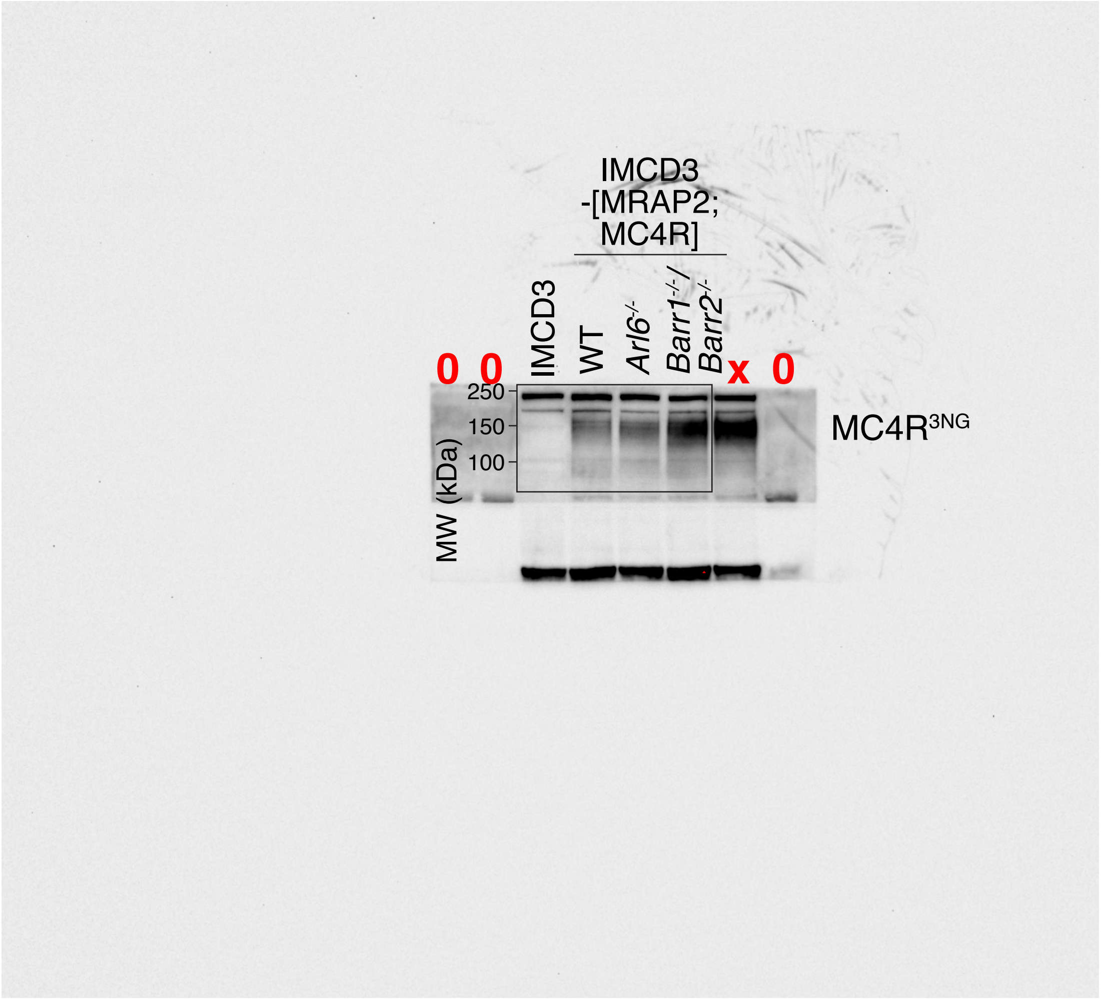

Two different exposures of the blot shown in the **Fig. 6D**. The squared parts of the blot were used in the main figure.

The lane marked with an **x** was not shown in the main figure, and it corresponds to another sample of the *Barr1-/-/Barr2-/- IMCD3-[MRAP2;MC4R]* cell line

The lanes marked with **0** contain molecular weight markers (Precision Plus Protein Standards Dual Color; Bio-Rad)

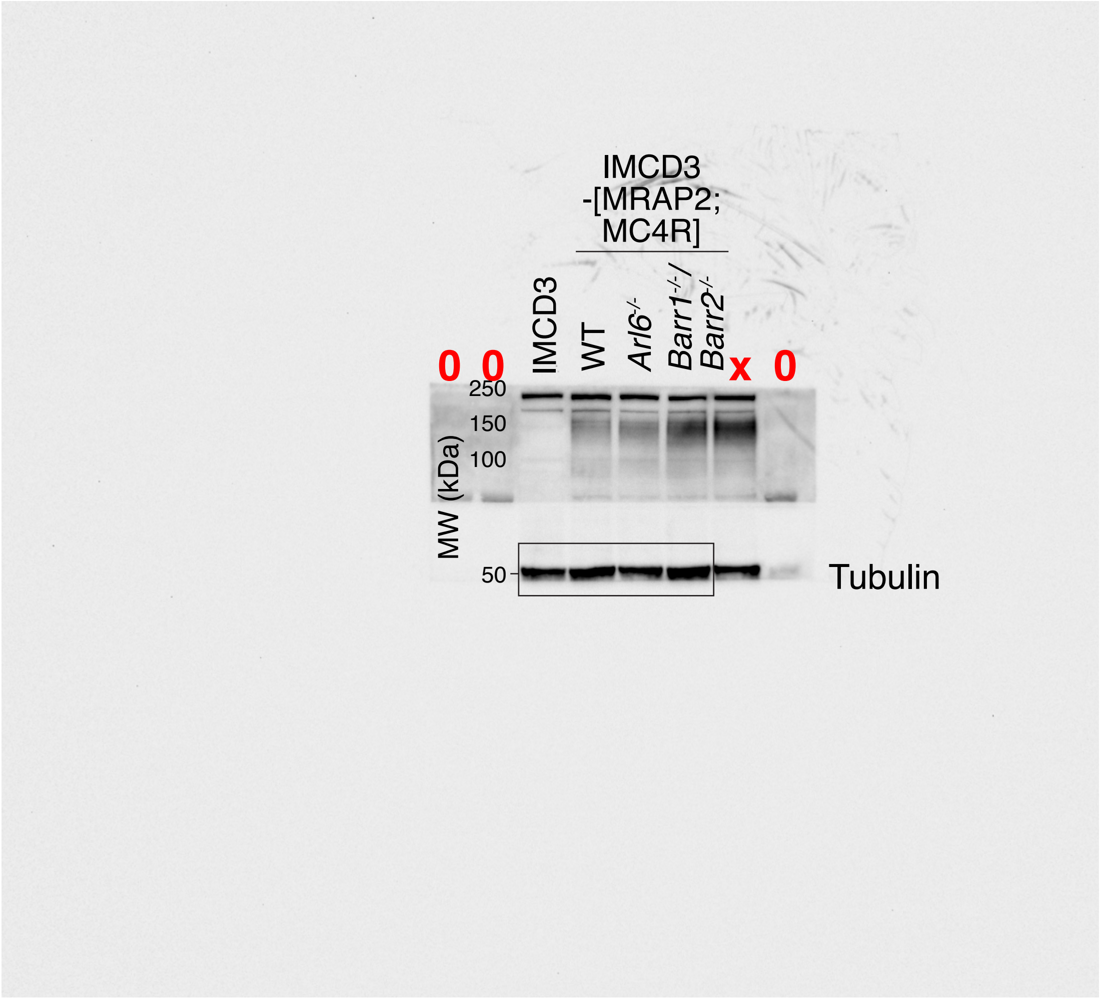

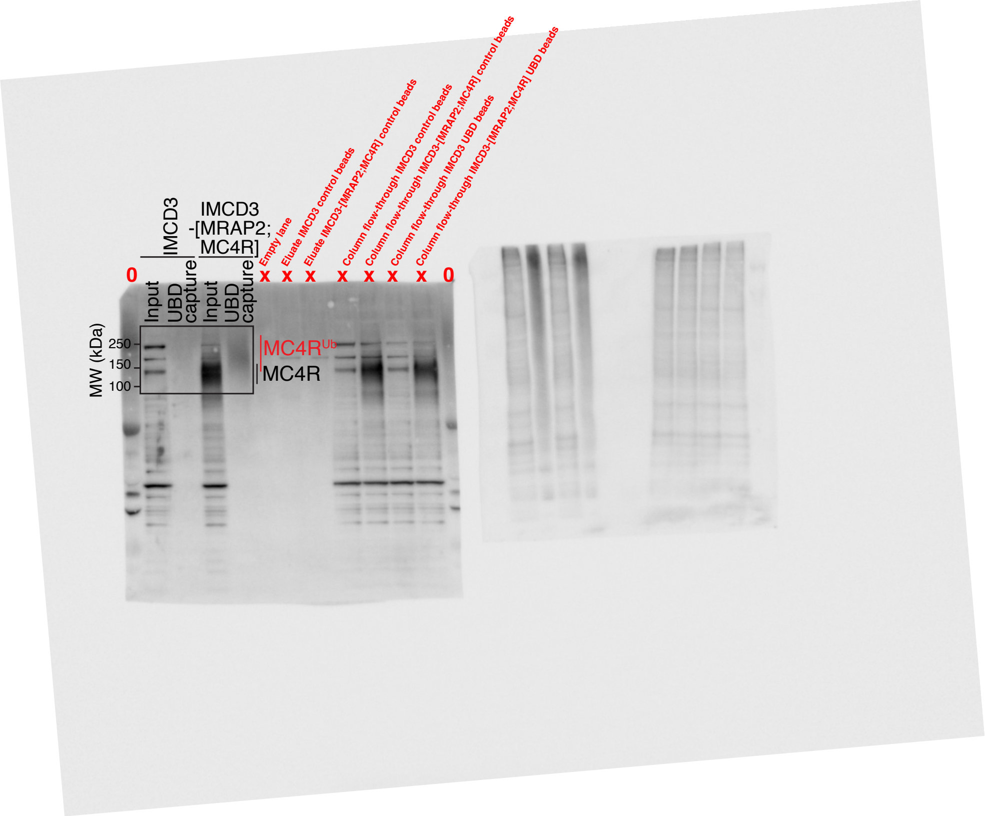

Two different exposures (top vs. bottom) of the blots shown in **Fig. 7A**. The squared parts of the blot were used in the main figure.

Bands appear red when the signal is saturated.

The blot on the left is probed for NeonGreen, the blot on the right is probed for ubiquitin (P4D1).

The lanes marked with an **x** were not shown in the main figure.

The lanes marked with **0** contain molecular weight markers (Precision Plus Protein Standards Dual Color; Bio-Rad)

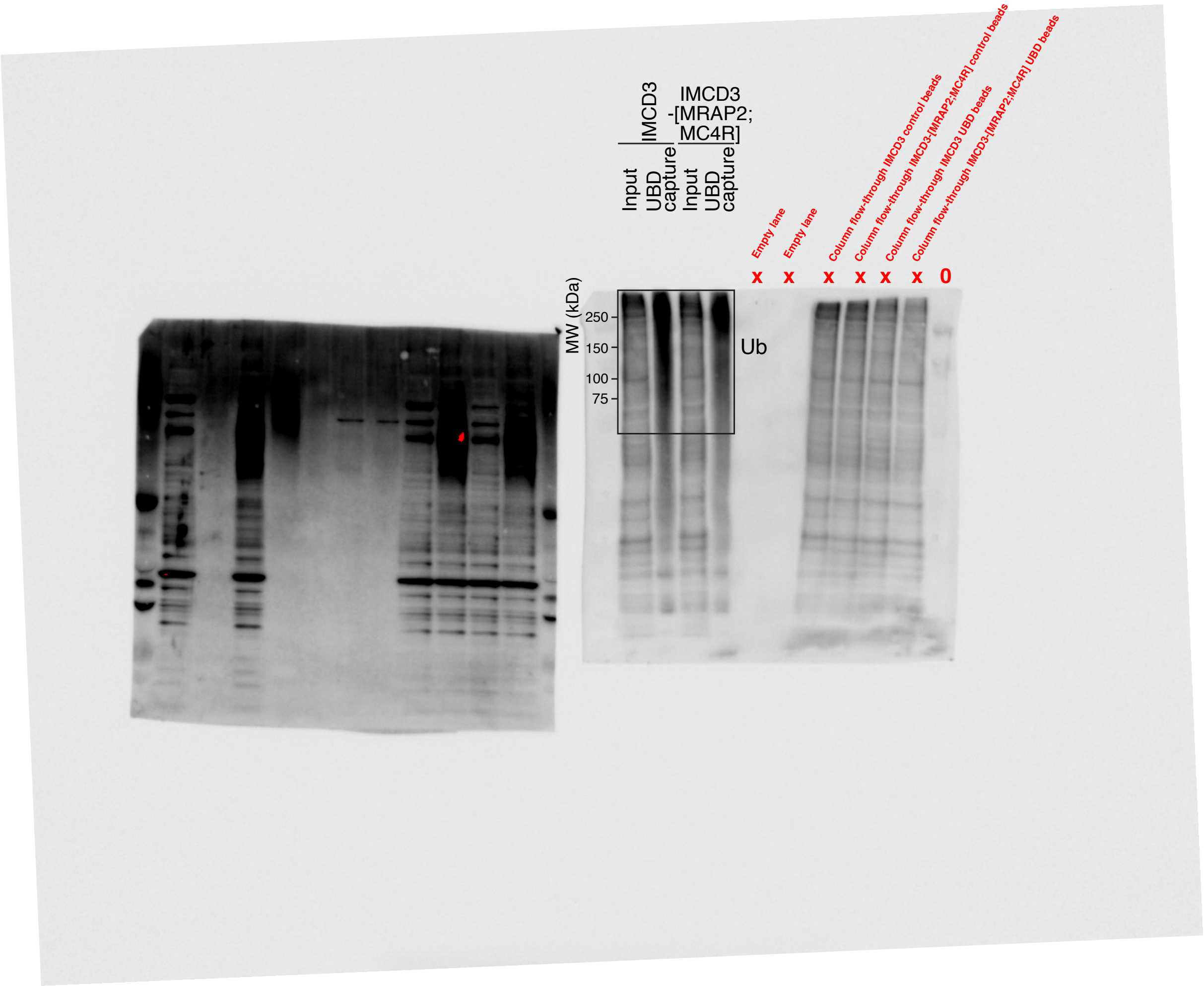

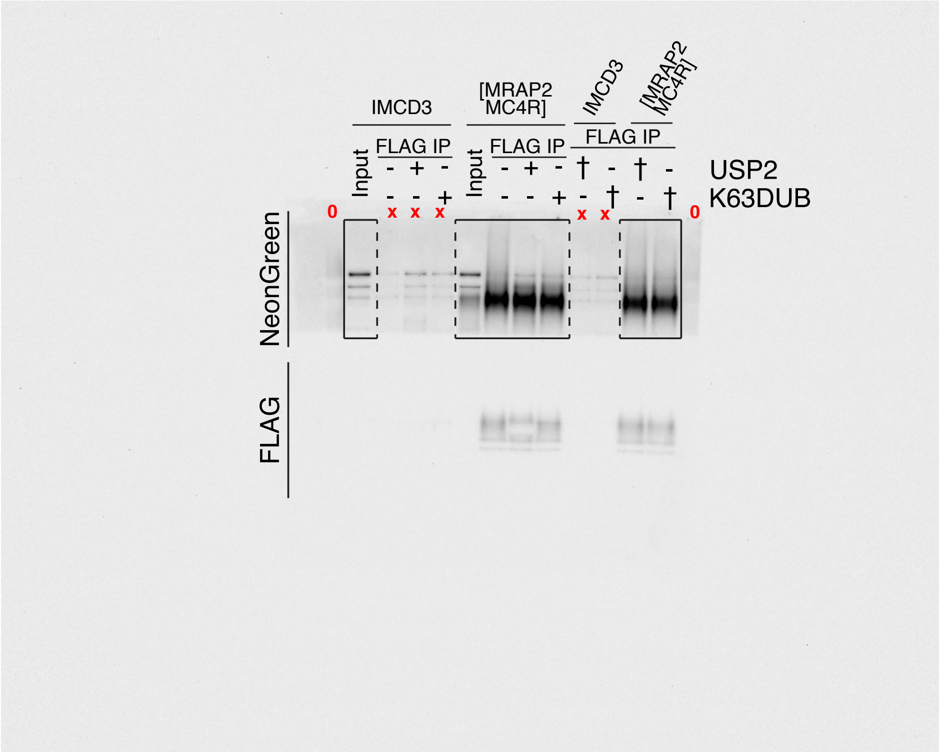

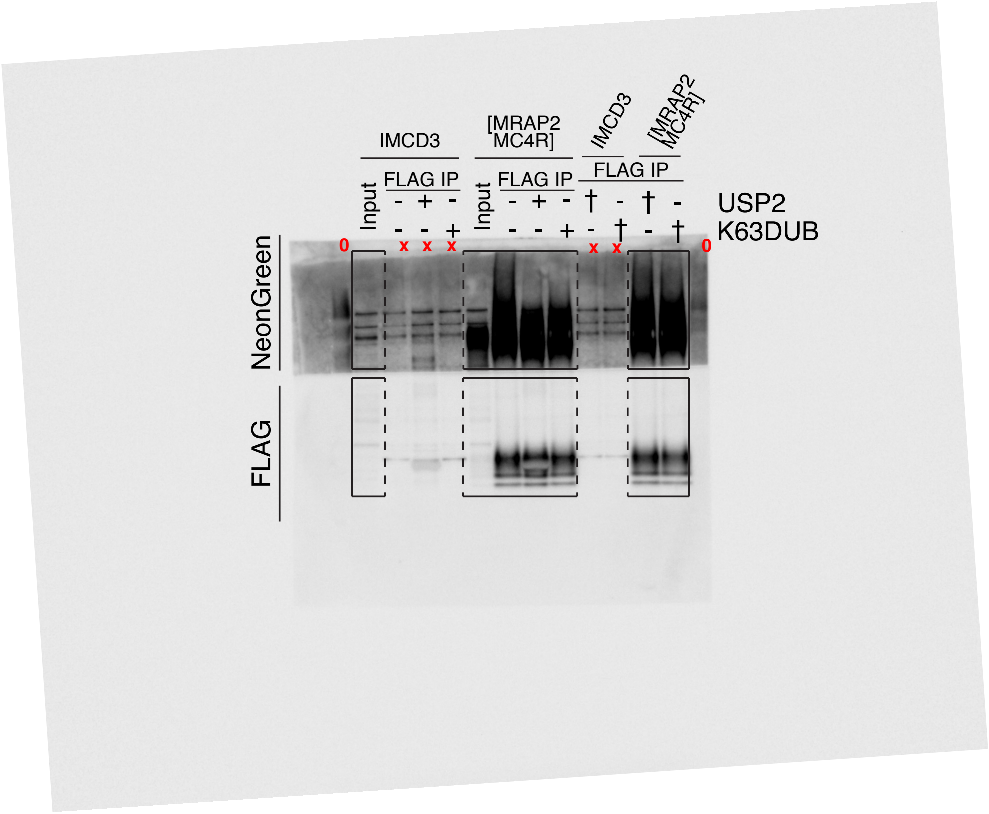

Two different exposures of the blot shown in **Fig. 7B**. The squared parts of the blot were used in the main figure. Dashed lines are used when the blot is partially cut.

The lanes marked with an **x** were not shown in the main figure.

The lanes marked with **0** contain molecular weight markers (Precision Plus Protein Standards Dual Color; Bio-Rad)

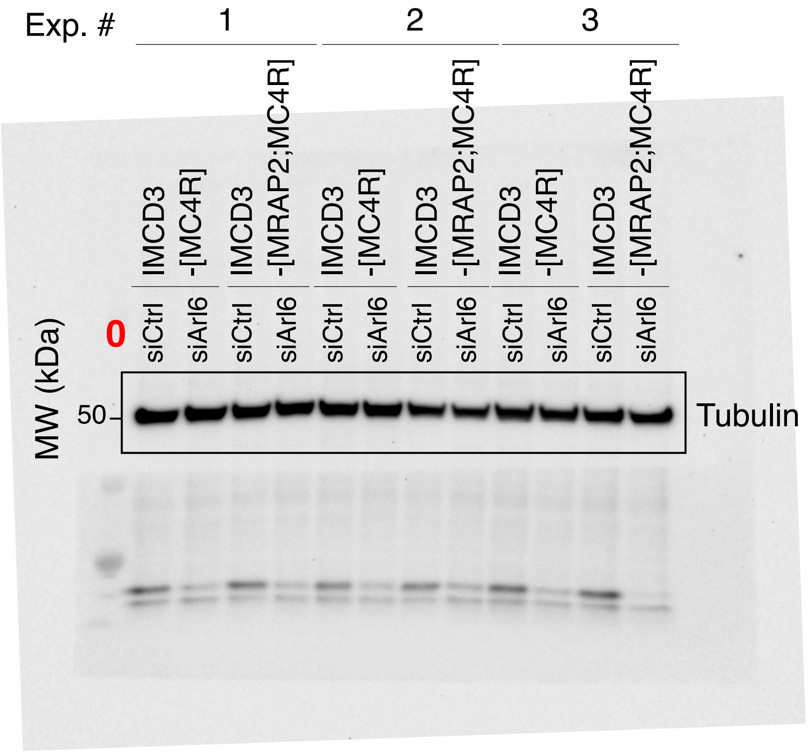

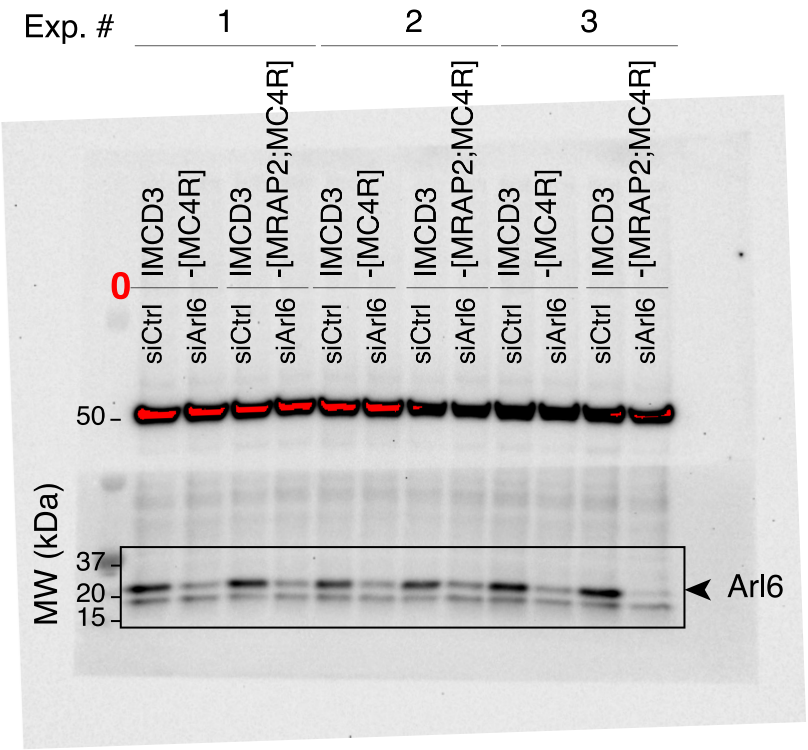

Two different exposures of the blot shown in **Fig. S3B**. The squared parts of the blot were used in the main figure.

Arl6 signal appears above 20 kDa, right above an unspecific band.

Bands appear red when the signal is saturated.

The lane marked with **0** contains molecular weight markers (Precision Plus Protein Standards Dual Color; Bio-Rad)
