# Supplementary material for: Tonic ubiquitination of the central body weight regulator melanocortin receptor 4 (MC4R) promotes its constitutive exit from cilia
Source: PLoS Biol. 2025 Feb 3;23(2):e3003025. doi: 10.1371/journal.pbio.3003025 (PMC11825094; doi:10.1371/journal.pbio.3003025)
Supplement: Attachment — Submitted filename: Response to reviewers_MN_FINAL.pdf [file pbio.3003025.s010.pdf]

We thank the reviewers and editor for their constructive critique of our manuscript. Most comments have been addressed in the revision. The new or modified text is highlighted yellow in the manuscript file.

REVIEWER 1

In this manuscript, the authors describe the mechanism mediating the constitutive exiting of MC4R from the primary cilium. The study is rigorous and novel. The data are compelling and support the proposed model.

We are grateful to the reviewer for the positive and constructive assessment of our study.

Minor comments:

1. The authors show that adding the MC4R antagonist AgRP results in MC4R ciliary accumulation. Is this reversed with co-treatment of AgRP and a-MSH to re-activate MC4R?

We have addressed this point by including a co-treatment assay with both ligands in our manuscript (**New Figure 3**). AgRP increases the ciliary levels of MC4R, and concomitant exposure to increasing concentrations of α-MSH decrease the ciliary levels of MC4R in a concentration-dependent manner. However, congruent with the potent antagonist activity of AgRP, α-MSH is unable to fully restore the ciliary levels of MC4R to baseline even when added at a 20-fold molar excess over AgRP.

2. The authors state that the fact that the accumulation of MC4R with AMSH (Figure 7B) is greater than with the MC4R-cKO (Figure 7E) suggests the presence of additional Ub acceptors. However, Figure 7E uses a non-clonal cell line with much higher basal ciliary MC4R expression than the isogenic cell line used in 7B, confounding this interpretation. Please also comment on why MRAP2-cK0 is not sufficient to prevent MC4R exit.

The reviewer is correct in pointing out the discrepancy between cell lines. We have now transfected cilia-AMSH (which we renamed cilia-K63DUB to remove any possible confusion between AMSH and α-MSH) and its controls in the $MRAP2_{cK0}/MC4R_{cK0}$ line. Cilia-AMSH can further increase the ciliary MC4R levels in this line, which offers more direct support for the existence of additional ubiquitin acceptors in the system besides MRAP2 and MC4R (**New Figure 8F-G**).

Our model posits that MC4R, MRAP2 and yet-to-be-defined interactors provide acceptor sites for the ciliary ubiquitination machinery. Removing some of the acceptor sites reduces the rate of exit from cilia, but a complete block of exit will require that all ubiquitin-acceptor sites be removed. Based on the magnitude of effects of the various cK0 mutants and of cilia-AMSH on ciliary exit, we believe that the cytoplasmic lysines of MRAP2 play a minor role in MC4R exit.

3. Please show protein levels of GPR161 and MC4R (Figure 1) to support the claim that MC4R is expressed at higher levels. Alternatively, if the tonic activity-mediated degradation complicates this analysis, provide transcript levels.

We acknowledge the need for empirical data to support the assumption that MC4R is expressed at higher levels than GPR161 in our cell lines. We have included a qRT-PCR assay, which shows that the transcript levels of $MC4R^{3NG}$ transcripts are 5-fold more abundant than $GPR161^{3NG}$ (**New Figure 1C**).

4. In the methods section, include the methodology for calculating the number of MC4R receptors in cilia.

We have added the requested information to the methods section. *The $GPR161^{3NG}$ line used as a calibrator for measuring the absolute number of $MC4R^{3NG}$ molecules per cilia was previously described in (Nager et al., 2017). The absolute number of ciliary $MC4R^{3NG}$ molecules was estimated by linearly interpolating the mean fluorescence value of all measured single cilia in the*

MRAP2/MC4R line (n=186), based on our previous calculation that the ciliary mean fluorescence value of the GPR161^{3NG} line (n=184 cilia in this study) is equivalent to 1,226 molecules.

5. In the discussion section, the paragraph discussing the role of GRK2 is too speculative given the data presented.
We have considerably shortened this paragraph to better align with the presented data.

6. Figure 3B - inset is in the wrong location
We have corrected the mistake in the **New Figure 4B**.

REVIEWER 2
Overall, I find that the paper is timely and well written, and the data quality is high. One could question the use of the IMCD3 cell model as opposed to a neuronal cell type, but on the other hand, the IMCD3 cell model used by the authors seems to be a "clean" system offering experimental control over relevant proteins and ligands. I have a few relatively minor comments/suggestions that the authors should address prior to publication of this work.
We wish to thank the reviewer for the words of support, in particular related to the advantages of the chosen model system.

Main comments
1) Figure 1, Figure S1 and 1st paragraph of results: western blots or RT-qPCR data should be provided for these cells to allow comparison of total cellular expression levels of the MC4R and GPR161 transgenes.
We acknowledge the need for empirical data to support the assumption that MC4R is expressed at higher levels than GPR161 in our cell lines. We have included a qRT-PCR assay to measure the transcript levels of GPR161^{3NG} and MC4R^{3NG} (**New Figure 1C**). We show that MC4R^{3NG} transcripts are 5-fold more abundant than GPR161^{3NG} transcripts.

2) Figure 2D, E: I am puzzled as to why the ciliary MRAP2-3FLAG levels increase so dramatically upon AgRP or HS014 treatment if ciliary entrance of MRAP2 alone does not rely on MC4R (Figure S5A-B; Bernhard et al., 2023)? Please comment.
This comment highlighted a need for improved clarity on the co-trafficking of MC4R and MRAP2. While MRAP2 can traffic to the cilium in the absence of MC4R, the interaction with MC4R increases its ciliary levels (**New Figure S4A-B**). Given that MRAP2 and MC4R form a complex, we propose that they co-traffic into and out of cilia. Thus, preventing the constitutive exit of MC4R from cilia quells the constitutive co-exit of MRAP2 from cilia. We have extensively revised the paragraph in the

To substantiate their model, it would be really nice if the authors could test how AgRP or HS014 treatment affects ciliary levels of MRAP2 alone, i.e. in the absence of MC4R.
Although MRAP2 is not expected to react to MC4R ligands, we directly addressed this point by transiently transfecting MRAP2^{3FLAG} into IMCD3 cells and concomitantly treating with inverse agonists or vehicle (**New Figure S4C-D)**. Neither AgRP nor HS014 modify the levels of ciliary MRAP2^{3FLAG} in the absence of MC4R.

3) In Figure S5, statistical analysis is missing and there is no indication about number of biological replicates. Also, are the total cellular expression levels of MRAP1-FLAG and MRAP2-FLAG identical in this setup?
In the New Figure S4, we included the statistical analysis of this experiment and clarified that we employed a single biological replicate. A nearly identical experiment was included in Figure S3 of

Bernard et al., 2023. To mitigate the inherent variability in expression levels of transiently expressed proteins, we have made our best effort to only quantify cells with low expression levels.

Very minor comment
1) Introduction, 9 lines from bottom: the word "syndromic" before "obesity syndrome" seems redundant and should be removed.
We have revised the style of this sentence and corrected it.

REVIEWER 3
The causal link between ciliary dysfunction and obesity is well established in human and animal models. GPCR MC4R is an important regulator of body weight homeostasis, and its ciliary localization has been shown (by the same group) to be key in this process. In this manuscript, Ojeda-Naharros and colleagues provided careful characterizations and convincing evidence that detailed the ciliary exit mechanisms of MC4R. Overall, the design of the study is elegant and thorough, and the quality of data is high.
We are grateful to the reviewer for the words of support.

This reviewer only has a couple of minor comments:

1. "SMO offers one exception…" paragraph in the discussion provides interesting insights regarding ciliary GPCR trafficking comparisons, but seems out of place since this paper is only focused on MC4R. It would be better to integrate the Smo information more tightly into the discussion about MC4R or to simply cut the paragraph.
We have cut this paragraph.

2. The importance of ciliary localization of MC4R in the prevention of obesity is hard to understand and is a key question to answer regarding the significance of MCR4 in obesity and ciliopathies. Although this question may be out of the scope of this study, a more thorough discussion (rather than only a few sentences in the end of the discussion) would be appreciated. The comment that "MC4R may reach its relevant physiological target immediately after exiting cilia, rather than inside cilia" seems interesting but difficult to understand because not much background information is provided. If MC4R has a strong tonic activity outside of cilia, why wouldn't the non-ciliary MC4R reach their targets, if the targets, as the author hinted, are localized near the base of cilia or the surrounding area in the cytoplasm?
We have edited this paragraph for clarity.
